# The metabolic response of human trophoblasts derived from term placentas to metformin

**DOI:** 10.1007/s00125-023-05996-3

**Published:** 2023-09-05

**Authors:** Jane L. Tarry-Adkins, India G. Robinson, Lucas C. Pantaleão, Jenna L. Armstrong, Benjamin D. Thackray, Lorenz M. W. Holzner, Alice E. Knapton, Sam Virtue, Benjamin Jenkins, Albert Koulman, Andrew J. Murray, Susan E. Ozanne, Catherine E. Aiken

**Affiliations:** 1 Department of Obstetrics and Gynaecology, the Rosie Hospital and NIHR Cambridge Biomedical Research Centre, University of Cambridge, Cambridge, UK; 2 Wellcome-MRC Institute of Metabolic Science and Medical Research Council Metabolic Diseases Unit, University of Cambridge, Addenbrooke’s Hospital, Cambridge, UK; 3 Department of Physiology, Neuroscience and Development, University of Cambridge, Cambridge, UK; 4 Centre for Trophoblast Research, University of Cambridge, Cambridge, UK

**Keywords:** Gestational diabetes mellitus, Metabolic, Metformin, Placenta, Pregnancy

## Abstract

**Aims/hypothesis:**

Metformin is increasingly used therapeutically during pregnancy worldwide, particularly in the treatment of gestational diabetes, which affects a substantial proportion of pregnant women globally. However, the impact on placental metabolism remains unclear. In view of the association between metformin use in pregnancy and decreased birthweight, it is essential to understand how metformin modulates the bioenergetic and anabolic functions of the placenta.

**Methods:**

A cohort of 55 placentas delivered by elective Caesarean section at term was collected from consenting participants. Trophoblasts were isolated from the placental samples and treated in vitro with clinically relevant doses of metformin (0.01 mmol/l or 0.1 mmol/l) or vehicle. Respiratory function was assayed using high-resolution respirometry to measure oxygen concentration and calculated ${\dot{V}\mathrm{O}}_{2}$. Glycolytic rate and glycolytic stress assays were performed using Agilent Seahorse XF assays. Fatty acid uptake and oxidation measurements were conducted using radioisotope-labelled assays. Lipidomic analysis was conducted using LC-MS. Gene expression and protein analysis were performed using RT-PCR and western blotting, respectively.

**Results:**

Complex I-supported oxidative phosphorylation was lower in metformin-treated trophoblasts (0.01 mmol/l metformin, 61.7% of control, *p*<0.05; 0.1 mmol/l metformin, 43.1% of control, *p*<0.001). The proton efflux rate arising from glycolysis under physiological conditions was increased following metformin treatment, up to 23±5% above control conditions following treatment with 0.1 mmol/l metformin (*p*<0.01). There was a significant increase in triglyceride concentrations in trophoblasts treated with 0.1 mmol/l metformin (*p*<0.05), particularly those of esters of long-chain polyunsaturated fatty acids. Fatty acid oxidation was reduced by ~50% in trophoblasts treated with 0.1 mmol/l metformin compared with controls (*p*<0.001), with no difference in uptake between treatment groups.

**Conclusions/interpretation:**

In primary trophoblasts derived from term placentas metformin treatment caused a reduction in oxidative phosphorylation through partial inactivation of complex I and potentially by other mechanisms. Metformin-treated trophoblasts accumulate lipids, particularly long- and very-long-chain polyunsaturated fatty acids. Our findings raise clinically important questions about the balance of risk of metformin use during pregnancy, particularly in situations where the benefits are not clear-cut and alternative therapies are available.

**Graphical Abstract:**

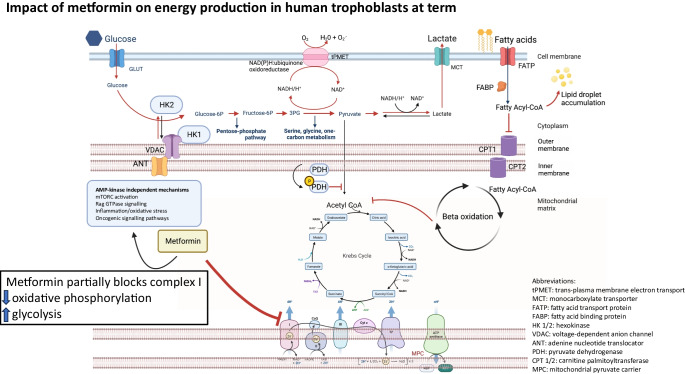

**Supplementary Information:**

The online version contains peer-reviewed but unedited supplementary material available at 10.1007/s00125-023-05996-3.


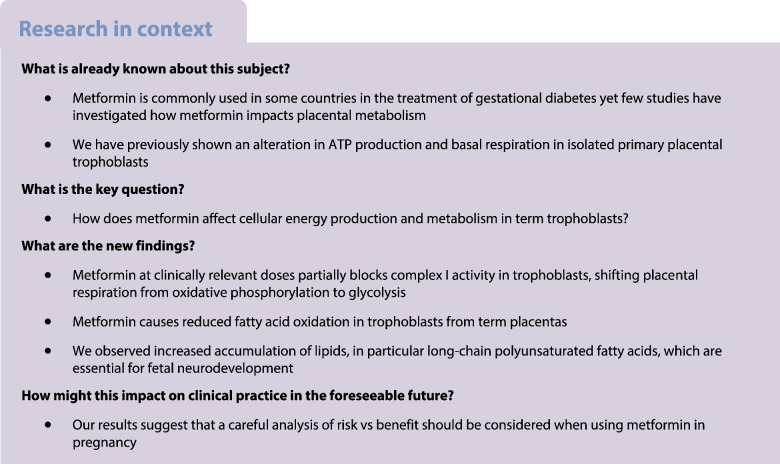


## Introduction

Metformin exposure during pregnancy is increasing worldwide. Hyperglycaemia, the most common reason for metformin treatment during pregnancy, affects as many as one in six pregnancies globally [1]. Data from the Metformin in the Women with Type 2 Diabetes in Pregnancy (MiTy) trial [2] show that metformin improves glycaemic control and reduces pregnancy weight gain in the context of type 2 diabetes in pregnancy. Other indications for which metformin is increasingly used during pregnancy include polycystic ovarian syndrome [3]. There is also promising evidence that metformin may be beneficial in treating severe early-onset pre-eclampsia [4], a major global cause of maternal and fetal morbidity and mortality, for which no other drug treatment is currently available [5]. In view of the high and increasing global rates of gestational diabetes and other indications for metformin therapy in pregnancy, it is likely that a considerable proportion of the next generation will be exposed to metformin whilst in utero. Previous studies have established that metformin can cross the placenta and is detectable in the fetal circulation [6, 7].

Metformin is a biguanide drug, most commonly prescribed for the treatment of type 2 diabetes [8, 9], and acts via various molecular pathways to produce tissue-specific effects (e.g. activating AMP-activated protein kinase [AMPK] [10], reducing oxidative stress [11], increasing circulating growth differentiation factor-15 (GDF15) [12] and potentially inhibiting mitochondrial electron transport chain activity [13]). However, in the highly metabolically active placenta, the net impact of metformin treatment is under-studied and remains unclear.

Metformin administered to a mother during pregnancy crosses the placenta and results in reduced offspring birthweight independent of maternal glycaemic control [14], possibly leading to catch-up growth, which confers an increased risk for childhood adiposity [3, 15]. A possible explanation for these findings is that metformin decreases placental ATP production [7], leading to reduced support for fetal growth (e.g. via reduced transfer of key nutrients). Hence, it is critically important that the implications of metformin treatment for placental metabolic function are understood, especially in view of the high and rising numbers of pregnancies exposed to metformin treatment.

We address this important knowledge gap by assessing the direct impact of metformin on third-trimester placenta. We use an established model of primary human trophoblasts cultured from term placentas and treated in vitro with metformin at clinically relevant concentrations [7] to profile a range of metabolic consequences.

## Methods

### Placental sampling

Placentas were donated by women undergoing full-term elective Caesarean section in Cambridge UK who met the study inclusion and exclusion criteria (Table 1). Demographic characteristics of the donors (*n*=55), which were in keeping with those of the maternity population as a whole, are presented in Table 2. Informed consent was obtained by a research midwife who collected the placentas immediately after birth and transferred them to the laboratory for immediate processing.

**Table 1:** Inclusion and exclusion criteria

Inclusion criteria	Exclusion criteria
Caesarean section with no prior labour	Multiple pregnancy
	Known major fetal anomaly
	Severe pre-eclampsia
	Any form of diabetes in pregnancy
	<37 weeks gestation
	Treatment with metformin during pregnancy

**Table 2:** Demographics data

Variable	Mean (IQR) or *n* (%)
Maternal age, years	34 (32–38)
Maternal BMI, kg/m^2^	28.4 (24–31)
Maternal weight category	
BMI ≤25 kg/m^2^	20 (36)
BMI >25 kg/m^2^	35 (64)
Sex of baby	
Female	30 (55)
Male	25 (45)
Placental weight, g	617.5 (540–682)
Birthweight, g	3637.4 (3265–3872)
Birthweight centile	72 (52–92)

### Isolation of primary trophoblasts

For all experiments, primary cytotrophoblasts were isolated from freshly collected placental tissue using a DNase digestion and centrifugation methodology, described in detail elsewhere [7]. Cells were plated in culture medium (electronic supplementary material [ESM] Table 1) at various densities (ESM Table 2) and were allowed to differentiate into syncytiotrophoblasts. Trophoblasts were cultured at 37°C under 5% CO_2_ and atmospheric oxygen. Cells were cultured for 96 h (4 days) before treatments. Cells were treated with metformin from 96 h (day 4) to 120 h of culture (day 5), then harvested at 120 h. Metformin concentrations were based on previous in vivo measurements during pregnancy [7]. The control group was treated with deionised water (1:1000 vol/vol). The medium was changed every 24 h.

### Mitochondrial respiratory function

Trophoblasts were trypsinised (TrypLE; Thermo Fisher Scientific, Waltham, USA), centrifuged and then re-suspended in warmed mitochondrial respiration medium (miR-05 solution; ESM Table 1) at 2×10^6^ cells/ml. Trophoblast suspensions were added to pre-calibrated chambers of an Oxygraph-2K (Oroboros Instruments, Innsbruck, Austria) at 37°C and stirred (750 rev/min), as described previously [16]. Cells were permeabilised with 10 µg/ml digitonin before commencing a substrate-uncoupler-inhibition titration assay (detailed in ESM Table 3). Initially, malate (2 mol/l) and pyruvate (25 mol/l) were added to support LEAK state respiration, followed by ADP (10 mol/l) to stimulate pyruvate-supported oxidative phosphorylation (OXPHOS). Next, glutamate (10 mol/l) was added to saturate complex I-supported respiration, followed by cytochrome *c* (0.01 mol/l) to assess outer mitochondrial membrane integrity and, finally, rotenone (0.5 µmol/l) to inhibit complex I. Between additions, respiratory rates were allowed to stabilise. Respiration rates were corrected for cell number. Data were processed using DatLab (version 6.1; Oroboros Instruments).

### Cell media glucose and lactate levels

Trophoblast cell culture medium was collected and centrifuged to remove debris. Glucose levels were measured using the QPulse glucose assay (Siemens Healthcare, Sudbury, UK) and lactate levels were measured using the Siemens Lactate Assay (Siemens Healthcare). Assays were conducted at the MRC MDU Mouse Biochemistry Laboratory, Cambridge, UK.

### Glycolytic rate and glycolytic stress assays

Glycolytic rate, glycolytic stress and non-mitochondrial respiration in trophoblasts were assayed using the Agilent Seahorse XF-96 Extracellular Flux Analyser (Agilent Technologies, Oxford, UK) according to the manufacturer’s suggested protocols (Wave Software v2.6, Agilent Technologies). Cell culture media was replaced with freshly prepared XF media (ESM Table 1). For the glycolytic rate assay, rotenone (mitochondrial complex I inhibitor 0.5 mol/l) or antimycin A (mitochondrial complex III inhibitor 0.5 mol/l) and 2-deoxy-d-glucose (2-DG 50 mol/l) were added. For the glycolytic stress test, glucose (100 µmol/l), oligomycin (100 µmol/l) and 2-DG (500 µmol/l) were added. The mito stress assay was conducted as described previously [7]. Representative examples of each assay are shown in ESM Fig. 1.

### Fatty acid uptake and oxidation assays

Cultured trophoblast cells were washed with PBS, then cultured in fresh, low-glucose DMEM containing 0.1 μCi/ml [1-^14^C]oleic acid (PerkinElmer, Waltham, MA, USA) with 0.0025% fatty acid-free BSA(wt/vol) for 1 h. The medium was transferred to scintillation tubes containing scintillation fluid. Cells were washed three times in ice-cold 0.1% BSA (wt/vol) solution in PBS and lysed in RIPA buffer. Cell lysates were centrifuged (10 min/16,000 *g*) and the supernatant fractions were added to scintillation fluid. Disintegrations per minute from [1-^14^C]oleic acid were measured in both the cell media and supernatant fractions using a TRI-CARB 5110TR Liquid Scintillation Counter system (PerkinElmer).

Fatty acid oxidation was measured as described previously [17]. Trophoblast cells were incubated in low-glucose DMEM containing 12.5 mol/l HEPES, 0.3% fatty acid-free BSA (wt/vol), 1 mol/l l-carnitine, 100 µmol/l oleic acid and 0.4 μCi/ml [1-^14^C]oleic acid. Filter paper discs saturated with 1 M NaOH were fixed to the inside of sealed wells and cells were incubated at 37°C for 3h. NaOH saturated disks were then carefully removed and added to scintillation fluid. Disintegrations per minute from 1-^14^C-labelled CO_2_ converted to sodium salts in the filter disks were measured using a TRI-CARB 5110TR Liquid Scintillation Counter system (PerkinElmer). All results were normalised to protein content and the mean of four technical replicates was reported for each sample.

### Trophoblast lipid content

Trophoblast cells were fixed with 4% buffered paraformaldehyde. Fixed cells were stained with diluted Nile Red solution (Sigma; 1 µg/ml), then incubated at room temperature, protected from light for 5 min. After washing, fluorescence was measured using a plate reader (emission *λ* 585 nm, excitation *λ* 515 nm; Tecan, Reading, UK) (ESM Fig. 2).

### Lipidomic analysis

Lipids were isolated from trophoblasts using an adapted liquid–liquid extraction protocol [18]. Briefly, chloroform–methanol (2:1) was added and cells were homogenised. Internal standards of lipid (1–10 µmol/l in methanol) and acylcarnitine (5 µmol/l in methanol) were added, and the mixture was homogenised. Following centrifugation (21,000 *g*/5 min), the organic lower layer (lipid extract) was removed and dried down using a ConcentratorPlus system (Eppendorf, Stevenage, UK) at 60°C for 180 min. Samples were reconstituted in propan-2-ol, acetonitrile and water (2:1:1 ratio) for LC-MS analysis. Full chromatographic separation of intact lipids used Waters Acquity H-Class HPLC System with the injection of 10 µl onto a Waters Acquity UPLC (CSH C18 column; 1.7 µm i.d., 2.1 mm×50 mm) maintained at 55°C. ESM Table 4 details the different phases used. The mass spectrometer (Exactive Orbitrap; Thermo Fisher Scientific) was calibrated immediately before sample analysis using a positive and a negative ionisation calibration solution (Thermo Fisher Scientific). The mass spectrometer scan rate was set at 4 Hz, giving a resolution of 25,000 (at 200 m/z) with a full-scan range of m/z 100–1800.

### Gene expression analysis

RNA was extracted from trophoblasts, quantified using a Nano-drop spectrophotometer (Thermo Fisher Scientific) and checked for integrity by running an agarose gel to check for the presence of the 18S and 28S ribosomal RNA bands (ESM Fig. 3), then synthesised to cDNA as detailed previously [19]. Gene expression was determined by RT-PCR using custom-designed primers (Merck, Darmstadt, Germany; ESM Table 5), which were designed to fall within one exon and run against human gDNA standard curves to determine copy numbers. SYBR Green PCR master mix (Applied Biosystems, Waltham, MA, USA) was used as previously described [19]. Equal efficiency of the reverse transcription of RNA from all groups was confirmed through quantification of expression of the housekeeping gene B2M, the expression of which did not differ between groups (vehicle, 5,143,615±943,441 copies; 0.01 mmol/l metformin, 4,998,756±760,223 copies; 0.1 mmol/l metformin, 5,153,399±556,920 copies).

### Protein expression analysis

Protein was extracted from trophoblasts as described previously [20]. Protein (20 µg) was loaded onto 10% polyacrylamide gels, electrophoresed, transferred to polyvinylidene difluoride membranes and detected using a variety of primary antibodies (ESM Table 6). Anti-rabbit IgG secondary antibodies were used (1:5000; Jackson Laboratories, Bar Harbor, ME, USA). Original blot images are shown in ESM Fig. 4. All antibodies were validated with positive and negative controls. All antibodies were diluted with 2% BSA (wt:vol) in Tris-buffered saline with Tween 20. Band densitometry was determined using chemiluminescence detection and Alpha Ease Imaging Software v4.1.0 (Alpha Innotech, Watford, UK).

### Statistical analyses

Data were analysed using hierarchal mixed linear regression models, with fixed effect for treatment group and random effect for placenta of origin. Each biological sample was treated with each experimental condition. No randomisation was required due to the triad structure of the data. Researchers were blinded to experimental groups during all experiments. Demographic factors (Table 1) were tested as covariates but these were not retained in our models as they made no difference to the results. Modelling results are presented as *β*±SEM. Numeric data are represented as mean±SEM. In graphical representations, box plots show median and IQR, with whiskers representing 1.5×IQR. Where *p* values are reported, *α*<0.05 was pre-specified as statistically significant. Data analysis was conducted using R v4.2.2 [21].

### Ethics approval

Study-specific ethics approval was granted under REC21/SC/0025 ‘The impact of metformin exposure on placenta ageing, metabolism and mitochondrial function’. Fresh placental collections were obtained via the biobanks of the Centre for Trophoblast Research (REC17/EE/0151) and from the Cambridge Blood and Stem Cell Biobank (REC18/EE/0199).

## Results

### Respiratory function

**Fig. 1:**
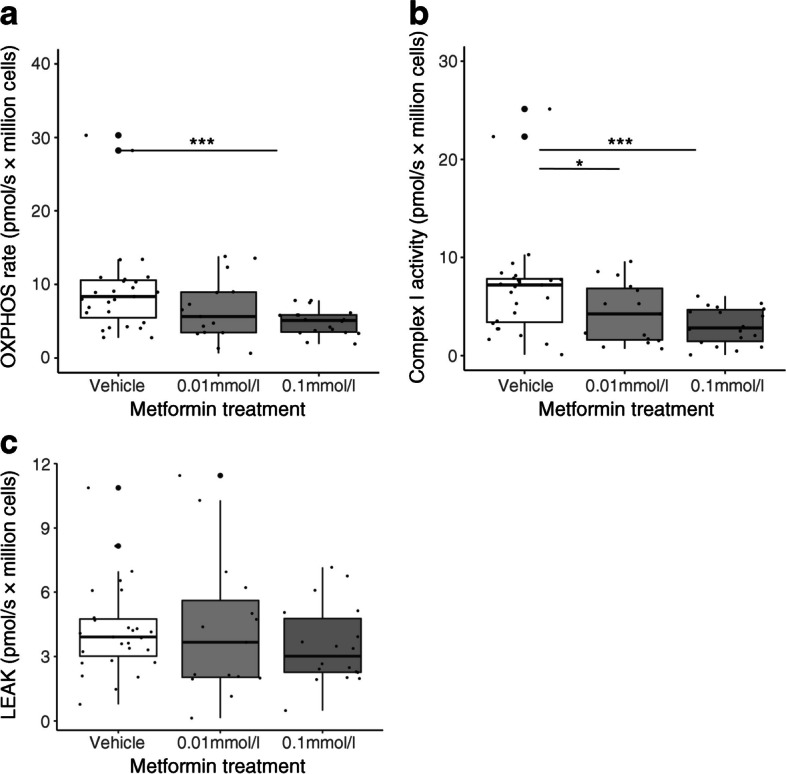
OXPHOS in human trophoblasts cultured with metformin. (**a**) Rate of OXPHOS (respiration rate when mitochondria are saturated with pyruvate, malate, ADP and oxygen). (**b**) Complex I activity (rate of respiration when complex I is maximally stimulated minus the rate of respiration when complex I is inhibited). (**c**) LEAK rate (basal oxygen flux that compensates for proton leak, proton slip, cation cycling and electron leak). Control group *n*=14; 0.01 mmol/l metformin *n*=12; 0.1 mmol/l metformin *n*=15. Technical replicates are plotted where applicable to display the total data distribution, but *n* refers to the number of placental samples. *p<0.05, ***p<0.001. Box plots represent median with IQR, and whiskers the max/min values excluding outliers, i.e. Q1/3±1.5×1QR

We initially assayed respiratory function in trophoblasts exposed to either 0.01 mmol/l or 0.1 mmol/l metformin for 24 h vs vehicle. Using high-resolution respirometry, we directly measured oxygen concentration and calculated ${\dot{V}\mathrm{O}}_{2}$ following exposure to a bespoke substrate-inhibitor-uncoupler titration. Pyruvate-supported OXPHOS was lower in trophoblasts treated with 0.1 mmol/l metformin than in trophoblasts treated with vehicle (control) (*p*<0.001); the OXPHOS was also lower in trophoblasts treated with 0.01 mmol/l metformin although this did not reach statistical significance (*p*=0.23; Fig. 1a). OXPHOS specifically supported by complex I was assessed by the rate of ${\dot{V}\mathrm{O}}_{2}$ following addition of glutamate to maximally stimulate complex I minus the rate of ${\dot{V}\mathrm{O}}_{2}$ when complex I was inhibited by the addition of rotenone. Complex I-supported OXPHOS was lower in both groups of metformin-treated trophoblasts compared with vehicle-treated controls (0.01 mmol/l *p*<0.05; 0.1 mmol/l *p*<0.001, Fig. 1b). Complex I-supported OXPHOS in the experimental groups was reduced to 61.7% (0.01 mmol/l metformin) and 43.1% (0.1 mmol/l metformin) of the control value. There was no significant difference in the LEAK rate between treatment and control groups (Fig. 1c), indicating that the differences observed were not due to metformin-induced alterations in proton leak or other components of basal state respiration.

### Glycolytic rate and glycolytic capacity

**Fig. 2:**
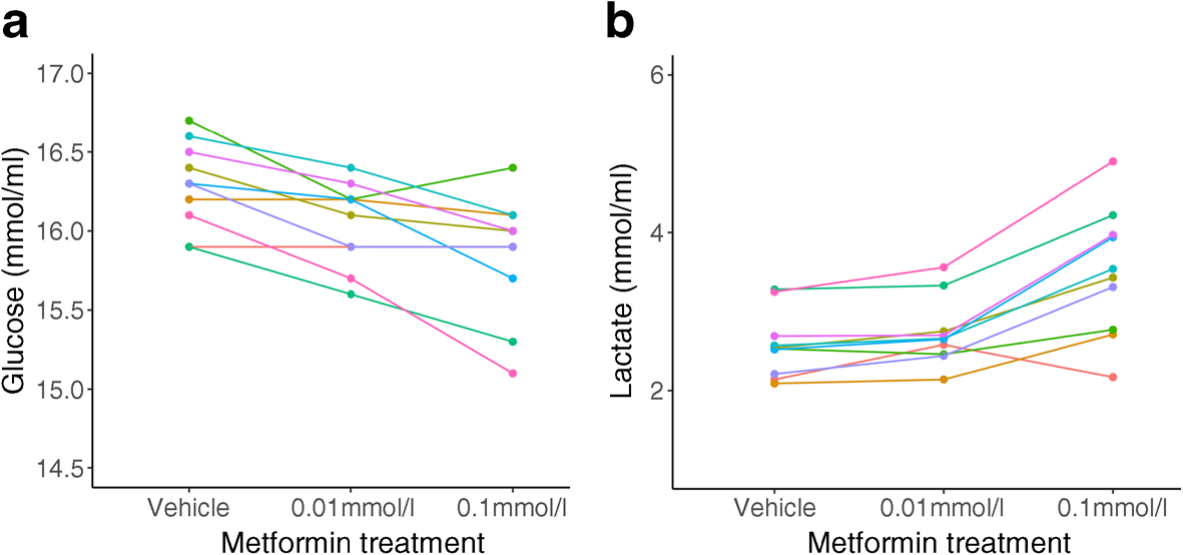
(**a**) Glucose concentration in trophoblast cell culture medium following 24 h treatment with vehicle, 0.01 mmol/l metformin or 0.1 mmol/l metformin. (**b**) Lactate concentration in cell culture medium following 24 h treatment with vehicle, 0.01 mmol/l metformin or 0.1 mmol/l metformin. Difference between groups *p*<0.001 for all comparisons. *n*=10 placental samples (each shown in a different colour) per group with a single technical replicate for each

In view of reduced OXPHOS in trophoblasts following metformin treatment, we next explored whether there might be a compensatory increase in glycolysis to maintain cellular energy production. Glucose concentrations in the trophoblast culture medium were reduced following treatment with both 0.01 mmol/l (*p*<0.001) and 0.1 mmol/l metformin (*p*<0.001) (Fig. 2a). Lactate accumulation increased following treatment with both 0.01 mmol/l (*p*<0.001) and 0.1 mmol/l metformin (*p*<0.001) (Fig. 2b), strongly suggesting dose-dependent upregulation of glycolysis in response to metformin treatment.

**Fig. 3:**
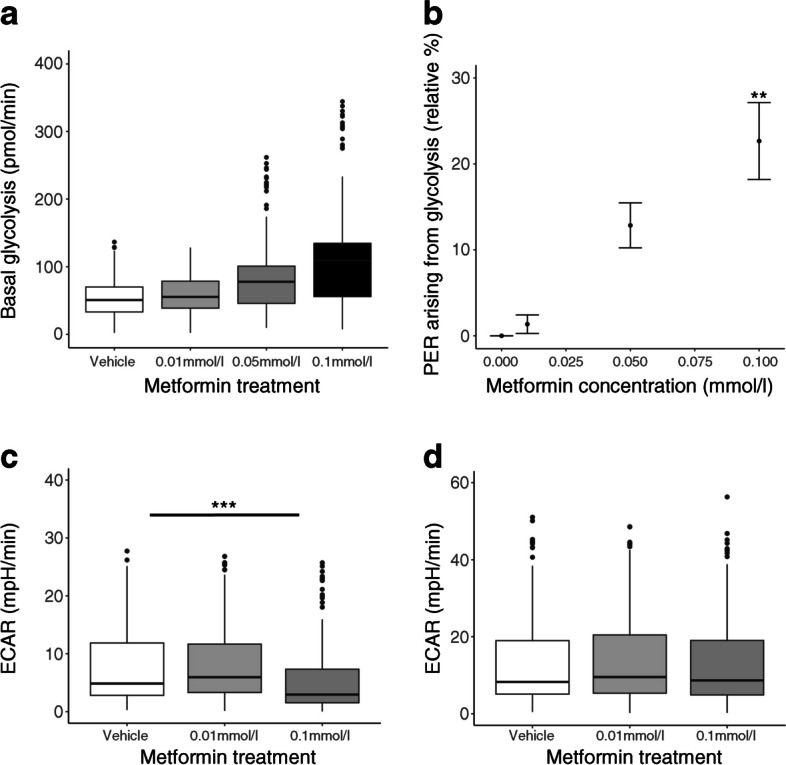
Glycolysis in human trophoblasts cultured with metformin. (**a**) Basal rate of glycolysis derived from ${\dot{V}\mathrm{O}}_{2}$ rate and extracellular acidification rate under basal conditions minus acidification due to mitochondrial activity. (**b**) Proton efflux rate arising from glycolysis expressed as a percentage of the control values. (**c**) Glycolytic reserve (difference between maximal glycolytic capacity and basal glycolytic rate). (**d**) Glycolytic capacity expressed as the maximal extracellular acidification rate following inhibition of mitochondrial ATP production minus the non-glycolytic acidification rate. **p<0.01, ***p<0.001. *n*=7 placental samples for each group, each of which was run in 16 technical replicate wells. Box plots represent median with IQR, and whiskers the max/min values excluding outliers, i.e. Q1/3±1.5×1QR. ECAR, extracellular acidification rate; PER, proton efflux rate

We explored glycolysis in metformin-treated trophoblasts further by measuring glycolytic rates under both physiological and stress conditions using Seahorse XF assays. There was a dose-dependent increase in the basal rate of glycolysis following metformin treatment (Fig. 3a). The proton efflux rate arising from glycolysis under physiological conditions was increased following metformin treatment, up to 23±5% above control conditions following treatment with 0.1 mmol/l metformin (*p*<0.01; Fig. 3b). Glycolytic reserve was reduced in metformin-treated trophoblasts compared with controls (0.01 mmol/l, non-significant; 0.1 mmol/l, *p*<0.001; Fig. 3c) glycolytic capacity was not affected by metformin (Fig. 3d). This suggests that metformin treatment causes trophoblasts to operate at a higher level within their glycolytic range rather than altering their overall potential to perform glycolysis.

Alongside our finding of increased rates of glycolysis, the expression of genes encoding placental glucose transporters (*GLUT1*, *GLUT3*, *GLUT4*, also known as *SLC2A1*, *SLC2A3*, *SLC2A4*, respectively) and hexokinases (*HK2*, *HK3* and *HK4*, also known as *GCK)* were unchanged following metformin treatment (Table 3). However, *HK1* expression was significantly reduced following treatment of the trophoblasts with 0.1 mmol/l metformin (*p*<0.001), consistent with reduced shuttling of substrate into the mitochondrial matrix [22]. Levels of phosphorylated pyruvate dehydrogenase (PDH) protein increased following treatment with 0.1 mmol/l metformin compared with vehicle (*p*<0.05), indicating PDH inhibition, but the increase did not reach statistical significance following treatment with 0.01 mmol/l metformin (*p*=0.30; ESM Table 7). There was no difference between groups in total PDH. Greater phosphorylation (inactivation) of PDH is in keeping with channelling of pyruvate away from mitochondrial oxidation towards lactate production [23].

**Table 3:** Effect of metformin on gene expression of placental glucose transporters and hexokinase isoforms in human trophoblasts

Gene	0.01 mmol/l metformin	*p* value	*n*	0.1 mmol/l metformin	*p* value	*n*
*GLUT1*	170,670±33,412	0.77	29	191,679±47,195	0.65	28
*GLUT3*	3331±541	0.91	30	3499±547	0.65	30
*GLUT4*	2238±729	0.32	30	1249±360	0.07	29
*HK1*	21,382±1738	0.99	30	15,025±1364	<0.001	30
*HK2*	5052±975	0.48	31	4085±637	0.83	31
*HK3*	13,694±2487	0.97	31	11,582±1887	0.47	31
*HK4*	2380±998	0.77	30	1272±529	0.68	31

Copy number is displayed as *n*±SE. *n* represents number of biological replicates

*p* values are derived from mixed-effects linear regression analysis with treatment group included as a fixed effect and placenta of origin as a random effect; the vehicle-treated group acts as the referent

### Lipid uptake and oxidation

**Fig. 4:**
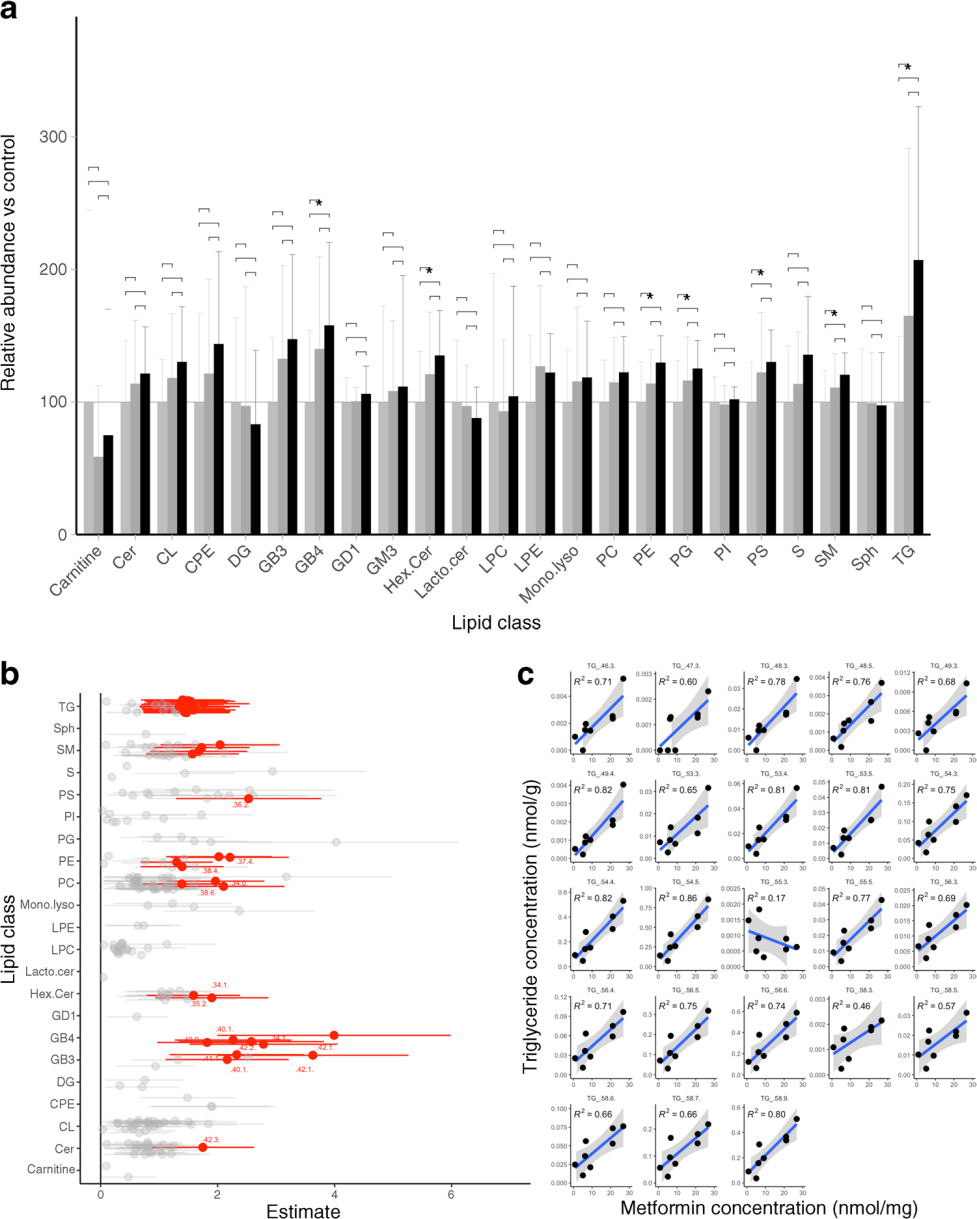
Lipid metabolism in human trophoblasts cultured with metformin. (**a**) Cellular lipid content by lipid class (normalised to vehicle-treated cells [control]). Values are shown as mean±SD for each group; *n*=10 in all groups. Light grey bars, control; dark grey bars, 0.01 mmol/l metformin; black bars, 0.1 mmol/l metformin; *p<0.05. (**b**) Forest plot showing relative abundance (OR±95% CI) of lipid species in 0.1 mmol/l metformin-treated trophoblasts vs control (vehicle-treated trophoblasts). Lipid species where *p*<0.05 following adjustment for false discovery rate are shown in red, *n*=10 per group. Analysis was adjusted for fetal sex, maternal BMI and gestational age at delivery. Unadjusted analysis is presented for comparison in ESM Fig. 5. (**c**) Metformin concentration vs triglyceride concentration for all triglyceride species likely to include long- or very-long-chain PUFAs. *R*^2^ for correlation is shown in each panel. Samples included where metformin level <2 SD from mean for either metformin concentration, *p*>0.01 for all species, except TG_48.5. *n*=8 placental samples per group with a single technical replicate for each. Cer, ceramide; CL, cardiolipin; CPE, ceramide phosphoethanolamine; DG, di-acylglyceride; GB, globotriaosylceramide; GD, disialodihexosylganglioside; GM, monosialodihexosylganglioside; Hex.Cer, hexosyl-ceramide; Lacto.cer, lactosyl-ceramide; LPC, lyso-phosphatidylcholine; LPE, lyso-phosphatidylethanolamine; Mono.lyso, mono-lyso-cardiolipin; PC, phosphatidylcholine; PE, phosphatidylethanolamine; PG, phosphatidylglycerol; PI, phosphatidylinositol; PS, phosphatidylserine; S, sulfatides; SM, sphingomyelin; Sph, sphingosines; TG, tri-acylglyceride

Having established that metformin causes the placenta to undergo a substantial shift away from OXPHOS and towards glycolysis for energy production, we next investigated how metformin impacts lipid uptake and oxidation. Initially using Nile Red staining on fixed primary trophoblasts, we detected a dose-dependent increase in lipid droplets in trophoblasts treated with metformin compared with vehicle control (0.01 mmol/l, *p*<0.05; 0.1 mmol/l, *p*<0.001; ESM Fig. 2). We investigated this result further using lipidomic analysis to determine which lipid species were accumulating in response to metformin treatment. There was a significant increase in abundance of seven out of 23 classes of lipids, including triglycerides, analysed following treatment with 0.1 mmol/l metformin (all *p*<0.05; Fig. 4a). In all the seven classes there was also an increase following treatment with 0.01 mmol/l metformin but the increase did not reach statistical significance. In no case was there a significant reduction in a lipid class following metformin treatment. Several individual lipid species increased in response to treatment with 0.1 mmol/l metformin, in both adjusted (Fig. 4b) and unadjusted analysis (ESM Fig. 5). These included individual species from nine different lipid classes, including several triglyceride and sphingomyelin species known to be important for fetal development. Triglyceride species were most commonly affected by metformin treatment, in particular those likely to be composed of long- and very-long-chain polyunsaturated fatty acids (PUFAs) (Fig. 4c). There was a clear positive dose–response relationship between metformin and the concentrations of these triglyceride species in primary trophoblasts (Fig. 4c, *p*<0.01 in 22/23 species with 0.1 mmol/l metformin; ESM Fig. 6 shows results with 0.01 mmol/l metformin).

**Fig. 5:**
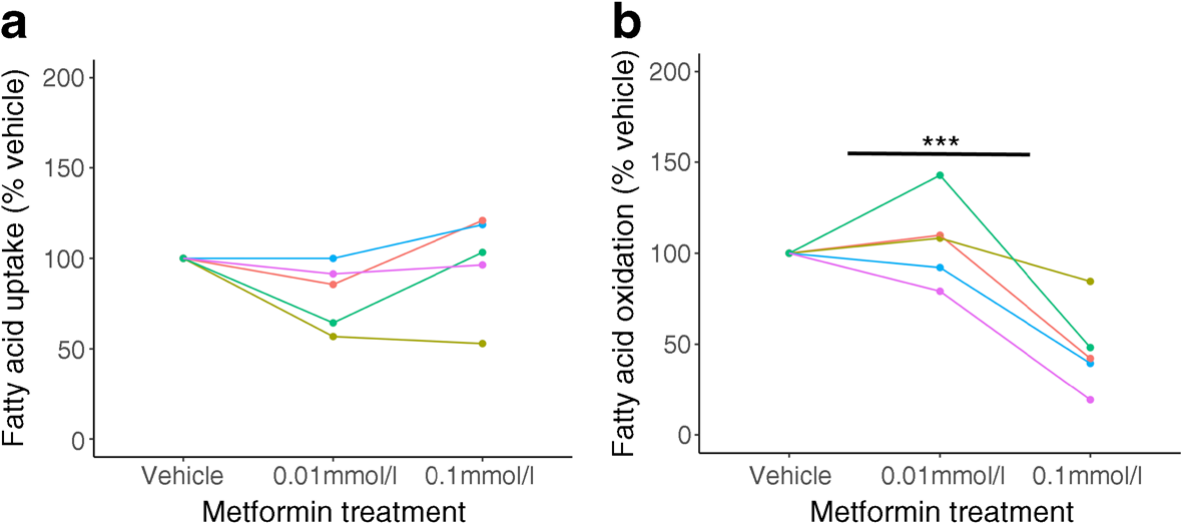
Lipid metabolism in human trophoblasts cultured with metformin. (**a**) Fatty acid uptake, expressed as percentage of vehicle-treated control; *n*=5 per group. (**b**) Fatty acid oxidation, expressed as percentage of vehicle-treated control. *n*=5 placental samples (each represented by a different colour), each of which was run in four technical replicates. ***p<0.001

Intracellular lipid accumulation could be the result of either increased uptake or reduced utilisation. We therefore performed dynamic assays on our primary trophoblast cultures using radiolabelled lipids to directly assess uptake and oxidation following metformin treatment. Fatty acid uptake from the cell culture medium was unaffected by metformin treatment at either concentration (Fig. 5a). Fatty acid oxidation was reduced by ~50% in trophoblasts treated with 0.1 mmol/l metformin compared with vehicle treatment (*p*<0.001) but the reduction did not reach statistical significance with 0.01 mmol/l metformin (*p*=0.16) (Fig. 5b; *n*=5 per group). Expression levels of genes encoding fatty acid transporters (*FATP1–5* [also known as *SLC27A1*–5]) were unchanged following treatment with 0.01 mmol/l metformin but *FATP1*, *FATP2* and *FATP4* were all significantly upregulated following treatment with 0.1 mmol/l metformin (Table 4). Increased fatty acid transporters may be required for increased formation and expansion of intracellular lipid droplets within the metformin-treated trophoblast [24].

**Table 4:** Effect of metformin on gene expression of fatty acid transporters and endothelial lipase in human trophoblasts

Gene	0.01 mmol/l metformin	*p* value	*n*	0.1 mmol/l metformin	*p* value	*n*
*FATP1*	3520±583	0.77	30	3841±572	0.04	30
*FATP2*	2195±303	0.91	30	3611±451	0.02	30
*FATP3*	2894±368	0.82	30	2889±364	0.65	30
*FATP4*	8377±876	0.92	32	11033±949	0.03	31
*FATP5*	821±195	0.77	32	679±137	0.91	32
*FATP6*	29,013±3748	0.99	32	34,663±4871	0.53	32
*LIPG*	8393±727	0.21	31	8001±1034	0.83	31

Copy number is displayed as *n*±SE. *n* represents number of placentas per group, run in duplicate

*p* values are derived from mixed-effects linear regression analysis with treatment group included as a fixed effect and placenta of origin as a random effect, with the vehicle-treated group as the referent

## Discussion

We found that in primary trophoblasts derived from term placentas metformin treatment caused a reduction in OXPHOS. Specifically, we demonstrated a reduction in complex I-supported respiration with an associated increase in glycolysis. Metformin-treated trophoblasts operate at higher basal glycolytic rates, closer to the ceiling of their glycolytic capacity. Fatty acid oxidation was also reduced in metformin-treated trophoblasts, likely as a consequence of metformin-induced partial blockade of the electron transport chain. Trophoblasts continue to take up fatty acids at the same rate in the presence of metformin, leading to intracellular fatty acid accumulation in lipid droplets following metformin treatment. Our findings are summarised in ESM Fig. 7.

Previous studies have suggested that metformin is an inhibitor of complex I in other tissues [25–27]; however, this has not previously been investigated in trophoblasts. Some studies suggest that metformin may only be a direct inhibitor of complex I at supratherapeutic concentrations, using concentrations over tenfold higher than those used in our experiments [28]. However, we have shown previously that the concentrations of metformin used in this study overlap with the concentration range measured in serum samples from mothers who were taking metformin therapy during pregnancy [7], suggesting that our finding of complex I inhibition is clinically relevant.

The shift from OXPHOS towards glycolysis for energy production in trophoblasts may have implications for placental function, particularly in meeting both the energetic demands of the third-trimester placenta itself and the energy required to support fetal growth. By the end of pregnancy, under physiological conditions the placenta produces 2.5 kg of ATP daily [29], which is necessary to fulfil its extensive biosynthetic roles in supporting both maternal adaptation to pregnancy and fetal development. We have shown previously that ATP production is reduced in metformin-treated trophoblasts [7], in keeping with a shift from OXPHOS towards glycolysis. Meta-analysis of clinical trials suggests that randomisation to metformin vs other treatments reduces birthweight independent of maternal glycaemic control in the context of gestational diabetes [15]. One potential explanation for this finding is an indirect effect of placental metformin exposure on fetal growth. It is possible that placental ATP production via glycolysis cannot keep pace with the energetic demands of supporting fetal growth thus leading to fetal growth restriction. This would be a clinically important finding, given the considerable short- and long-term risks associated with poor fetal growth in utero [30, 31]. Other potential interpretations include a direct effect of metformin on fetal growth, as it is known to cross into the fetal circulation at concentrations comparable with those in the maternal circulation [7].

The electron transport chain is a convergence point of various pathways of cellular metabolism [32]. Complex I inhibition therefore has multiple impacts, including reducing β-oxidation of fatty acids [33]. We show that metformin treatment reduces β-oxidation in trophoblasts by ~50%. As fatty acid uptake remains unaltered, there is significant intracellular accumulation of triglyceride species. We show that the specific triglycerides accumulating within metformin-treated trophoblasts are esters of long-chain PUFAs. In particular, the triglycerides identified in metformin-treated trophoblast are likely to be esters of arachidonic acid and docosahexaenoic acid (DHA), in addition to several sphingomyelin species, all of which are also essential for fetal central nervous system development [34]. Towards the end of pregnancy, fetal DHA requirements are very high (approaching 70 mg daily) as they are essential components of neural growth and development [35]. Under physiological conditions, DHA levels in the fetal circulation reflect those in the mother [36], although we show that PUFAs accumulate within metformin-treated trophoblasts. Therefore, investigating whether the fetal availability of PUFAs is reduced in metformin-exposed pregnancies is an important area of future research.

Strengths of our study design include the ability to compare the impact of clinically relevant doses of metformin in trophoblasts derived from the same donors. This circumvents questions about how unobserved variation (e.g. genetic factors [37]), maternal smoking or variation in trophoblast growth from different placentas may influence metformin response. The known demographic characteristics of our donors were tested and found not to contribute to inter-sample variation in metformin response. Furthermore, the availability of a large cohort of placentas delivered exclusively by elective Caesarean section increases confidence in our findings. We were also able to take account of the sex of the placentas as a co-variate in our models but did not find significant differences between male and female placentas in their response to metformin.

Our study design also has limitations. In particular we studied trophoblasts that were mainly differentiated in culture to syncytiotrophoblasts [38], whereas previous work suggests that cytotrophoblasts have different baseline glycolytic capacity and energy production [39]. We did not formally assess for any impact of metformin on syncytialisation or apoptosis. We modelled the effect of short-term metformin exposure using trophoblasts from full-term placentas that had already differentiated in culture. However, it is possible that there may be different impacts where trophoblasts are exposed to metformin during most or all of a pregnancy. We were also unable to directly model the fetal impacts of the placental changes we observe in our human trophoblast culture system. Animal models of metformin treatment in pregnancy may contribute to filling this important knowledge gap [40, 41], as will human studies that investigate potential longer-term impacts of metformin treatment on the offspring (e.g. growth [14] and neurodevelopmental outcomes [42]).

We show that metformin causes a substantial shift in trophoblast energy production from OXPHOS towards glycolysis, accompanied by the intracellular accumulation of triglycerides. A key feature of the trophoblast response to metformin treatment is the increased amount of glucose required to maintain glycolytic energy production. This may contribute to the efficacy of metformin as a treatment for gestational diabetes [43] via the absorption of additional glucose from the maternal circulation. Given the harms that result from untreated gestational diabetes [44], in situations where alternative treatments such as insulin are not feasible, metformin may be extremely useful to achieve maternal normoglycaemia and thus mitigate these risks [43]. Furthermore, metformin-induced suppression of complex I may result in decreased generation of reactive oxygen species in trophoblasts [7]. The resulting reduction in oxidative stress may underlie the observed efficacy of metformin in delaying delivery in the context of severe early-onset pre-eclampsia [4]. Severe early-onset pre-eclampsia remains a life-threatening condition, in which even small increases in gestation at birth can result in large gains in infant survival rate [45]. Therefore, although our results raise important questions about the long-term impacts of metformin exposure during key developmental phases, these are set against the benefits of metformin treatment in specific high-risk circumstances. However, our findings that metformin reduces energy production and causes trophoblasts to accumulate fatty acids bring into question whether it is an ideal drug in low-risk pregnancy or where alternative treatments are available. Further investigation of the complex impacts of metformin on the bioenergetic and anabolic functions of the placenta will allow more specific targeting of metformin treatment in pregnancy to individuals for whom the benefits outweigh the potential risks.

### Supplementary Information

Below is the link to the electronic supplementary material.

Supplementary file1 (PDF 1145 KB)

## Data Availability

Data are available upon request to the corresponding author.

## Abbreviations

2-DG: 2-Deoxy-d-glucose
DHA: Docosahexaenoic acid
OXPHOS: Oxidative phosphorylation
PDH: Pyruvate dehydrogenase
PUFAs: Polyunsaturated fatty acids
