# Supplementary material for: The metabolic response of human trophoblasts derived from term placentas to metformin
Source: Diabetologia. 2023 Sep 5;66(12):2320–31. doi: 10.1007/s00125-023-05996-3 (PMC10627909; doi:10.1007/s00125-023-05996-3)

ESM Table 1: Media components

A) Trophoblast culture media

Components
BenchStable DMEM/F12 (1:1) with GlutaMax containing sodium bicarbonate (2.438g/L) and sodium pyruvate (Cat no: A41920-01)
FBS (10% vol/vol)
Penicillin-Glutamine-Streptomycin (1% vol/vol)
Amphotericin B (0.1% vol/vol)
Gentamycin (0.1% vol/vol)

B) Glycolytic rate assay media

Components	Stock conc.	Volume (ml)	Working conc.
**XF Base media**	-	38.5	N/A
**Glucose**	2500mM	0.5	10mM
**Sodium pyruvate**	100mM	0.5	1mM
**L-glutamine**	200mM	0.5	2mM
**pH adjustment to 7.4 & H_20 top up**	-	1.5	N/A

C) miR-05 solution

Component	Quantity
**miR-05**	110 mM sucrose
	60 mM K-lactobionate
	20 mM HEPES
	20 mM taurine
	10 mM KH_2PO_4,
	3 mM $MgCl_2$
	0.5 mM EGTA
	% (w/v) fatty acid-free BSA
**H20**	230ml
**KOH (5M)**	3.75ml (pH 7.1)
**BSA**	0.25g

ESM Table 2: Cell plating densities used for different experimental techniques

Outcome measure	Cell density	Volume (ul)
Oxygraph	2,000,000	2000
RNA and protein	4,000,000	4000
Glycolysis Stress Test	500,000	80
Nile Red Staining	100,000	100
Fatty acid oxidation	1,000,000	500
Fatty acid uptake	1,000,000	500
Fatty acid secretion	1,000,000	500
Lipidomics	1,000,000	1000

ESM Table 3: Components used to measure complex I in SUIT assay (listed in order of addition)

Substrate/Uncoupler/Inhibitor	Volume (µl)	Final Conc. (mM)
Malate	10	2
Pyruvate	25	25
ADP	20	10
Glutamate	10	10
Cytochrome C	5	0.01
Rotenone	1	0.0005

ESM Table 4:

A Components of mobile phases used in the HPLC separation of lipids

Mobile Phase A	Mobile Phase B	Flow rate	Gradients
6:4 acetonitrile:H_20	9:1 Propan-2-o1: acetonitrile with 10mM ammonium formate	500ul/minute	0 min: 40% mobile phase B<br>1.5 min: 40% mobile phase B<br>8 min: 99% mobile phase B<br>12 min: 40% mobile phase B

B Sample injection needle washing details

Strong Wash	Weak wash
9:1 propan-2-o1:acetonitrile	2:1:1 propan-2-o1:acetonitrile:H_20

ESM Table 5: Primer table for gene expression studies

Gene	Sequence (F, 5'-3')	Sequence (R, 5'-3')	Product size (bp)
GLUT 1	TGAGCATCGTGGCCATCTTT	GAGGTCCAGTTGGAGAAGCC	142
GLUT 3	AAATTGGACCAGGCCCCATT	GAGGGGAAGAGCAATCCGAC	133
GLUT4	TGGGCTTCTTCATCTTCACC	CGTTCTCATCTGGCCCTAAA	64
HK 1	CTGAATAGCACCTGCGATGA	CCCACAGTCACATTCAGACG	152
HK 2	GAGGAGATGCGCAACGTG	GCCGGGGTTGAGTGAAAG	150
HK 3	TGATGGTGCTAGAGGTGTGC	GCTTGTAGAGCGTTCCATCC	150
HK 4	CCGCAAGCAGATCTACAACA	AGCTTGTACACGGAGCCATC	213
FATP-1	GGGTCAGATCAACCAACAGG	GAGAGGTAGGCGCTGTCG	117
FATP-2	CTGGTTTGCAAAATCACACAA	TCTCCAACTCTGTCGTGGAA	176
FATP-3	AGCAGAAAGTTCGGATGGCA	CAGTTGTGAGGGGCAGGTAG	105
FATP-4	ACAGAGCTACGGAAGGAGGG	CTGTAGGCCTCTTGGTCCAG	107
FATP-5	AGGGCTTCAATGTGGGGATC	CCTCACACACAGCCTGGTAC	105
FATP-6	GCATCAGTTGGTGGAAGATGG	AGTTCCCTGGTCAGTAGAACA	102
LIPG	GGTCTACACCGAGGAGGACT	ACAGGTAGCTGCGAAACTCC	107

ESM Table 6: Primary antibodies

Antibody	Concentration	Source	Catalogue number
Anti-phosphoPDH^{ser293}	1:1,000	Cell Signalling, Bar Harbor, Maine, USA	31866
anti-pyruvate dehydrogenase (PDH)	1:1,000	Proteintech, Rosemont, USA	18068-1-AP
anti-hexokinase-1 (HK-1)	1:1,000	Proteintech, Rosemont, USA	19662-1-AP
anti-hexokinase-2 (HK-2)	1:1,000	Proteintech, Rosemont, USA	22029-1-AP

ESM Table 7: Effect of metformin on protein expression of PDH and phosphorylated PDH in human syncytiotrophoblasts (0.01mM or 0.1mM). Biological replicates; n=8; technical replicates; n=1. Results are derived from mixed-effects linear regression analysis with treatment group included as a fixed effect and placenta of origin as a random effect, with the vehicle-treated group as the referent. Model estimates for each group are displayed with p values in brackets. P<0.05 in bold. A) Total PDH, B) Phosphorylated PDH.

**Protein**	**0.01mM met**	**0.1mM met**
Total PDH	-3790.50 (0.81)	17244 (0.30)
Phosphorylated PDH	1127.88 (0.92)	24717.63 **(0.03)**

ESM Fig. 1: Example data from A) glycolysis stress test, B) proton efflux rate (PER), C) proton efflux rate (% glycolysis; glycoPER), D) extracellular acidification rate (ECAR), E) Oxidative capacity rate (OCR)

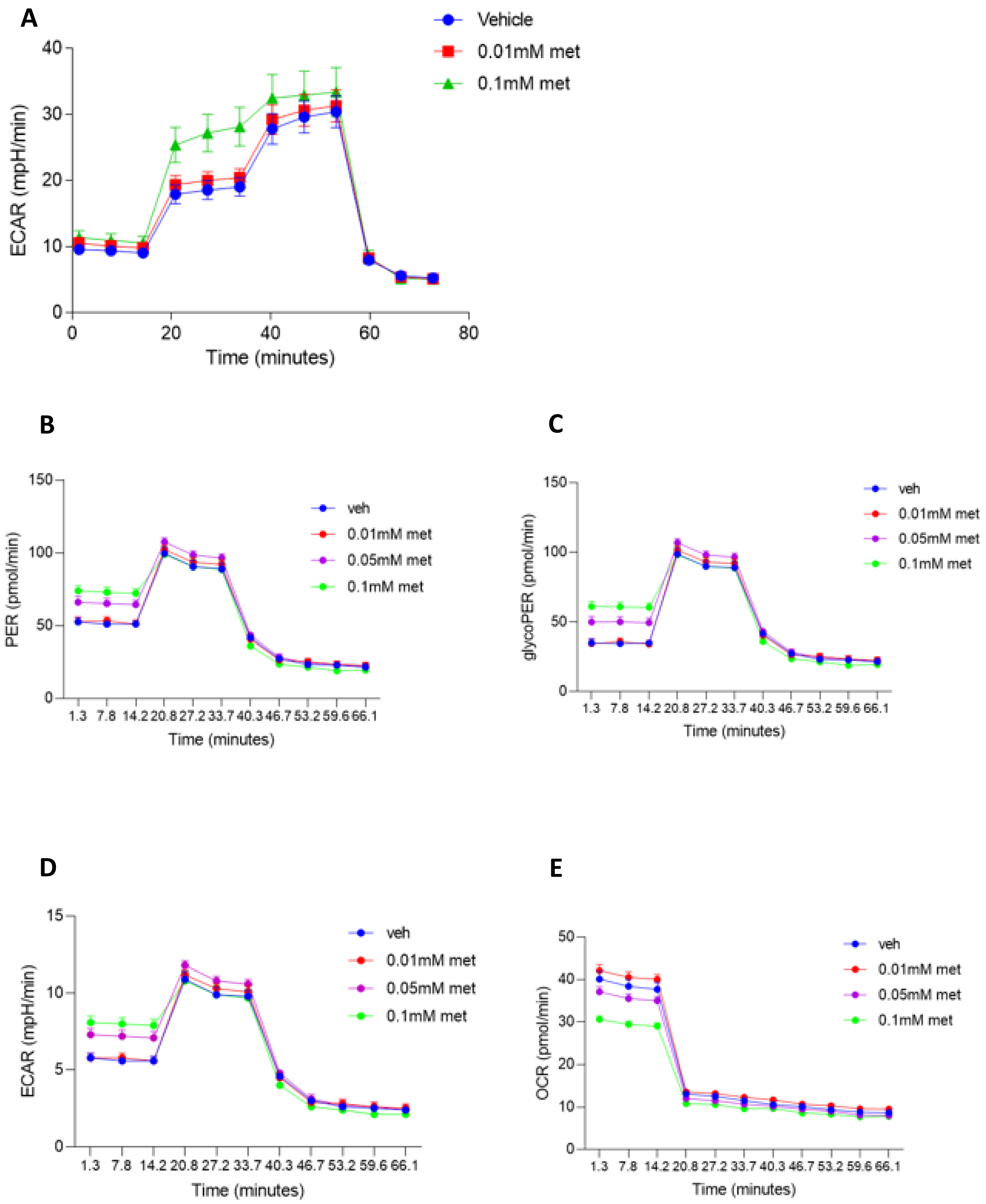

ESM Fig. 2: Effect of metformin treatment on lipid droplet accumulation in human syncytiotrophoblasts. Biological replicates: n=8; technical replicates: n=1

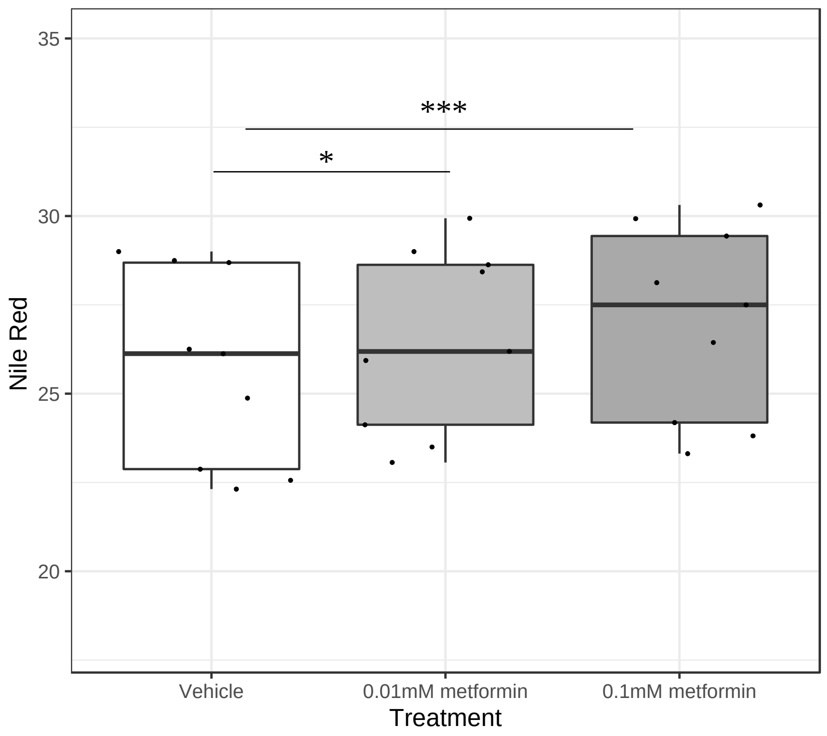

ESM Fig. 3: Representative agarose gel for demonstration of good integrity RNA used in this study. RNA isolated from primary human trophoblasts treated with metformin and control samples.

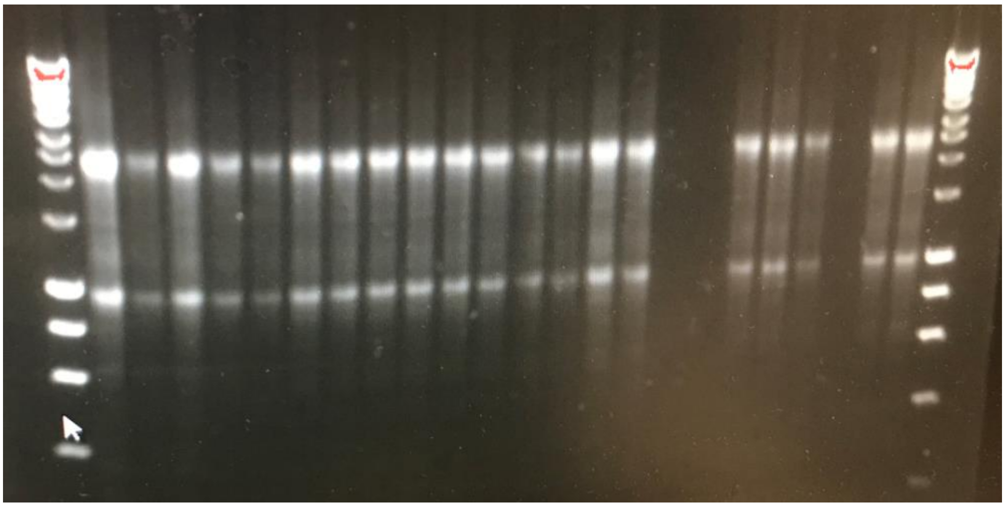

ESM Fig. 4: Representative protein blots for proteins used in this study. Protein derived from cultured human trophoblasts treated with 0.1mM, 0.01mM and control. (a) Total PDH, (b) Phosphorylated PDH.

A)

M +ve C M

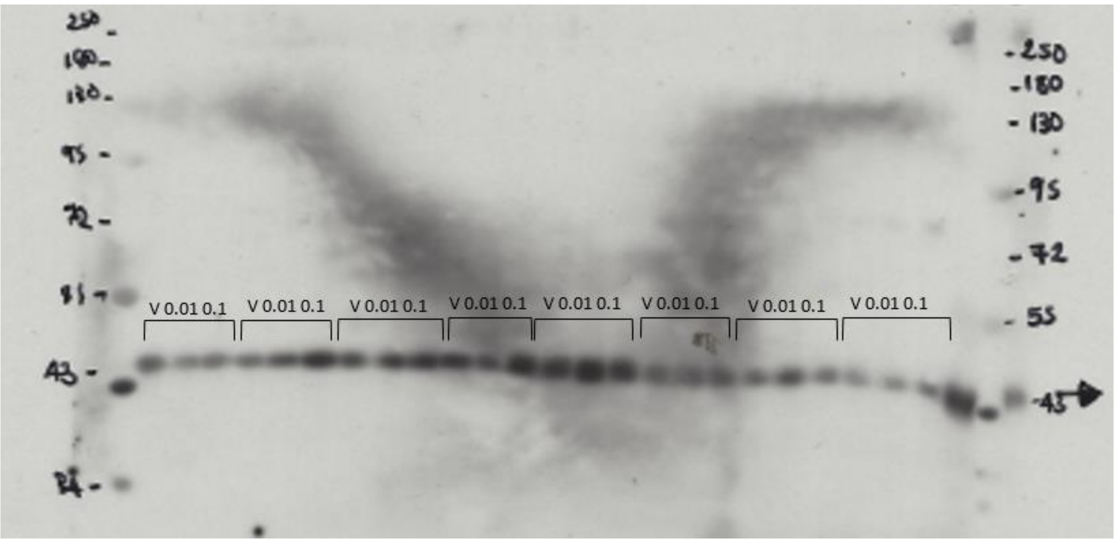

B)

M +ve C +ve C M

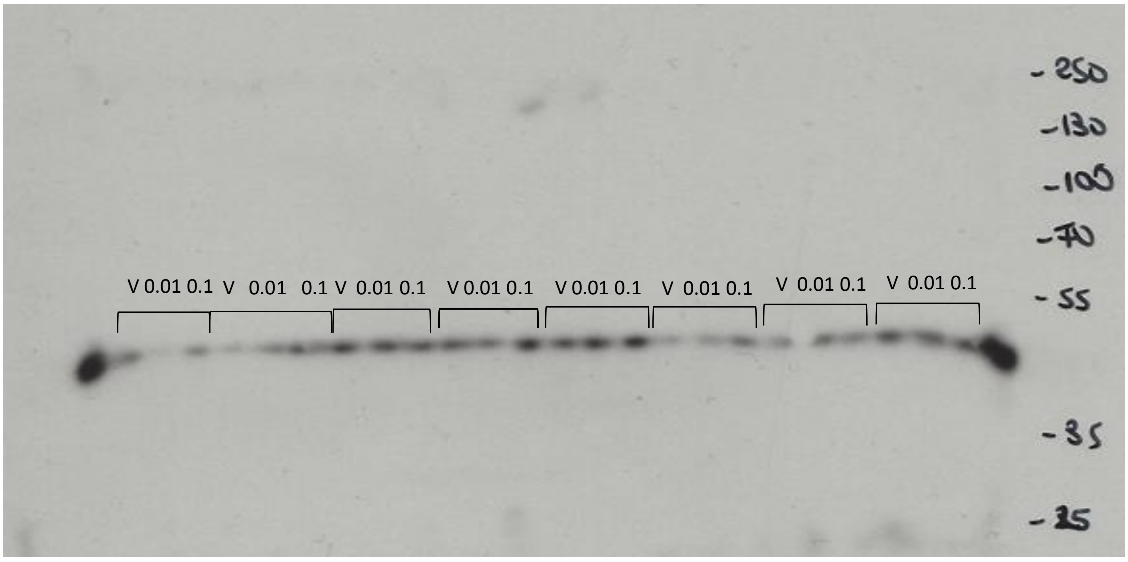

C) Representative Coomassie Blue protein loading, demonstrating equal loading of protein

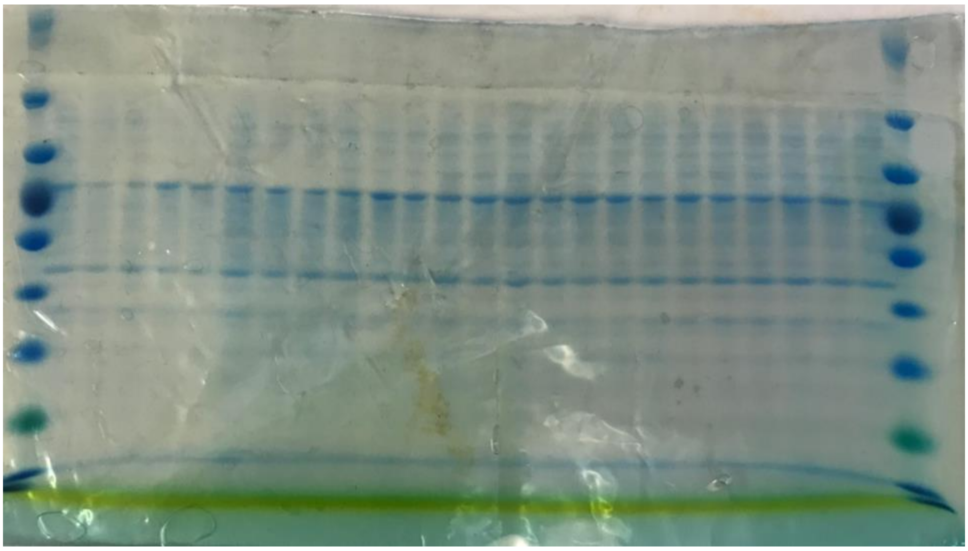

ESM Fig. 5: Effect of metformin treatment on lipid species in human syncytiotrophoblasts (0.1mM metformin relative to control; OR ± 95%CI, unadjusted analysis). Species significantly different to control (p<0.05 after adjustment for FDR) are shown in red. Biological replicates; n=8; technical replicates; n=1.

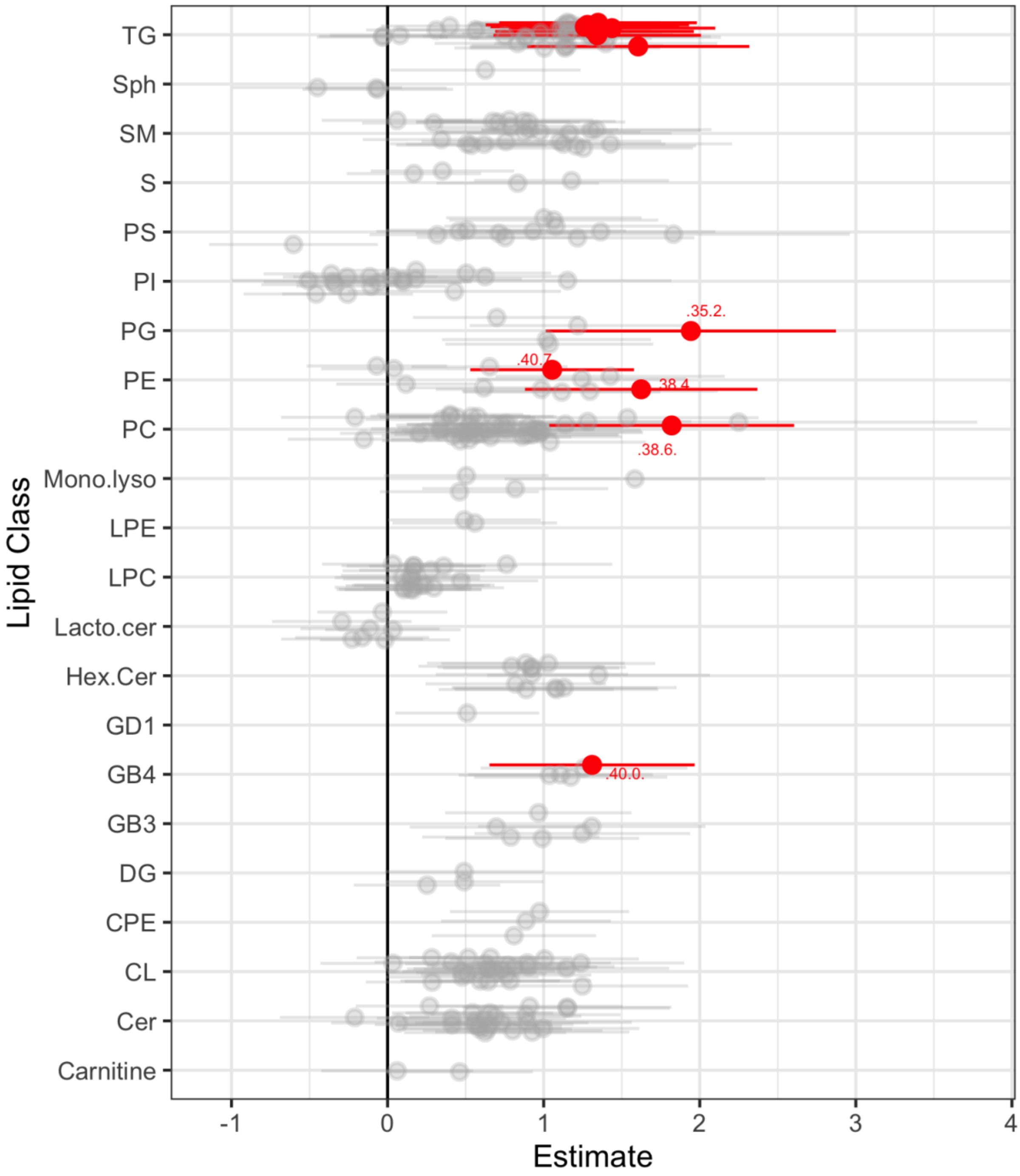

ESM Fig. 6: Metformin concentration v. triglyceride concentration for all triglyceride species likely to include poly-unsaturated long or very long chain fatty acids, measured in primary cultures of human syncytiotrophoblasts treated with 0.01mM metformin. R^2 for correlation shown in each panel. Biological replicates; n=8; technical replicates; n=1. (Samples excluded where metformin level >2SD from mean, with either metformin concentration). p >0.1 for all species, except TG 48_5 (p=0.057).

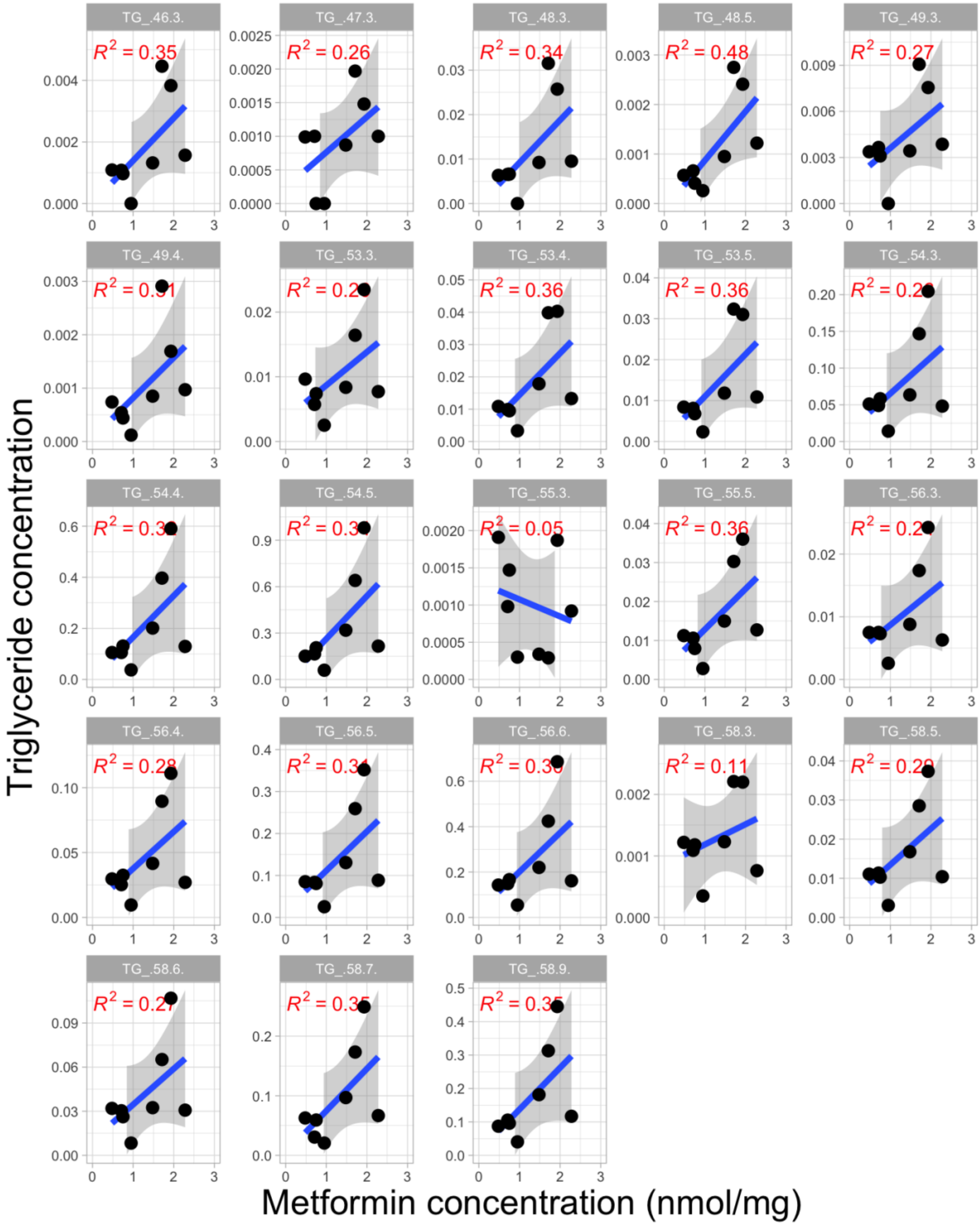

ESM Fig. 7: Summary of metabolic changes in response to metformin in human trophoblasts
tPMET: trans-plasma membrane electron transport
MCT: monocarboxylate transporter
FATP: fatty acid transport protein
FABP: fatty acid binding protein
HK 1/2: hexokinase
VDAC: voltage-dependent anion channel
ANT: adenine nucleotide translocator
PDH: Pyruvate dehydrogenase
CPT 1/2: carnitine palmitoyltransferase
MPC: mitochondrial pyruvate carrier

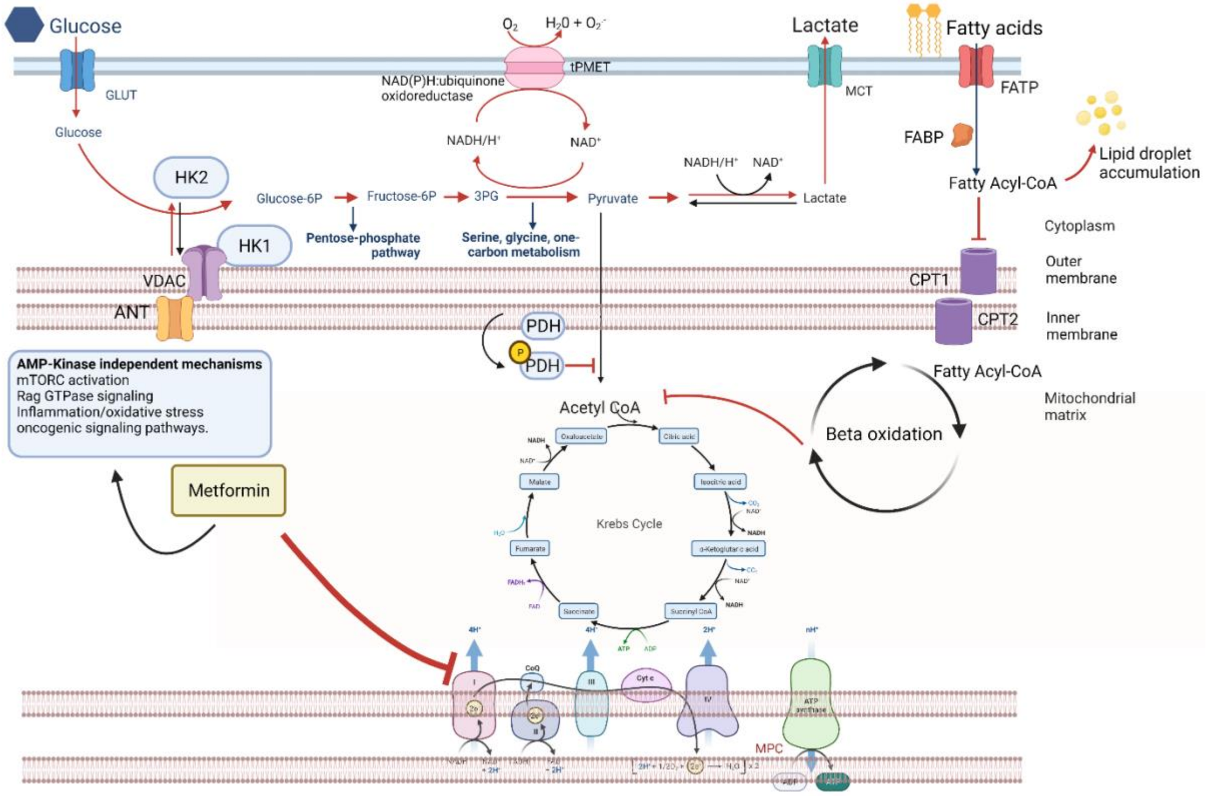

Supplement: Supplementary file 1 — Supplementary file1 (PDF 1145 KB) [file 125_2023_5996_MOESM1_ESM.pdf]
